# Screening Cover Crops for Utilization in Irrigated Vineyards: A Greenhouse Study on Species’ Nitrogen Uptake and Carbon Sequestration Potential

**DOI:** 10.3390/plants13141959

**Published:** 2024-07-17

**Authors:** Mehdi Sharifi, Khaled Salimi, Daniel Rosa, Miranda Hart

**Affiliations:** 1 Summerland Research and Development Centre, Agriculture and Agri-Food Canada, Summerland, BC V0H 1Z0, Canada; 2 Department of Agronomy and Crop Breeding, Faculty of Agriculture, Higher Educational Complex of Saravan, Saravan P.O. Box 9951634145, Iran; ksalimi@gmail.com; 3 Department of Biology, University of British Columbia Okanagan Campus, Kelowna, BC V1V 1V7, Canada; d.rosa@ubc.ca (D.R.); miranda.hart@ubc.ca (M.H.)

**Keywords:** grasses, forbs, legumes, nitrogen use efficiency, nitrogen utilization efficiency, plant biomass, wine grape

## Abstract

This study examines the potential of 23 plant species, comprising 10 legumes, 9 grasses, and 4 forbs, as cover crops to enhance carbon (C) sequestration and soil nitrogen (N) in vineyards. After a 120-day evaluation period, cover crop biomass was incorporated into the soil, and grapevine seedlings were planted in its place. Among the established cover crops, the C input potential ranged from 0.267 to 1.69 Mg ha^−1^, and the N input potential ranged from 12.3 to 114 kg ha^−1^. Legume species exhibited up to threefold greater shoot dry weight (SDW) compared to grass species. Ladino white clover, Dutch white clover, and Clover blend were superior in SDW, total dry weight (TDW), total C content, and total N content. Legumes exhibited slightly higher root dry weight (RDW) than grasses, with the exception of Fall rye leading at 15 g pot^−1^, followed by Ladino white clover and Dutch white clover at an average of 12 g pot^−1^. Legumes, particularly clover blend and Alsike clover, displayed high shoot N concentration at an average of 2.95%. Root N concentration in Legumes (Fabaceae) were significantly higher at 1.82% compared to other plant families at 0.89%, while their root C/N ratio was lower at 18.3, contrasting with others at 27.7, resulting in a faster turnover. Biomass production exhibited a negative relationship (R^2^ = 0.51) with soil residual NO_3_^−^. Fall rye, Winfred brassica, and buckwheat had the highest N utilization efficiency (NU_t_E) values (ava. 121 g g^−1^). Alsike clover, Ladino white clover, and clover blend showed the highest N uptake efficiency (NU_p_E) values (ava. 75%). The Readily Available N (RAN) Reliance Index (RANRI) is introduced as a novel indicator for quantifying the extent to which a plant relies on RAN for its total N requirement. The RANRI value represents the percentage of the plant’s total N sourced from RAN, ranging from 11% for legumes to 86% for grasses. This implies a substantial influx of nitrogen through a pathway independent of RAN in legumes. Grape shoot N concentration positively correlated with soil NO_3_^−^ (R^2^ = 0.31) and cover crop C/N ratio (R^2^ = 0.17) but negatively correlated with cover crop TDW (R^2^ = 0.31). This study highlights legume plants as more effective in C and N assimilation during establishment but cautions about potential soil mineral N depletion before reaching their full biological N fixation capacity.

## 1. Introduction

To sustainably support a projected population of 10 billion by 2050, agriculture must reduce dependencies on fossil fuels, fertilizers, and pesticides, while addressing environmental impacts such as soil fertility loss, biodiversity decline, erosion, organic matter depletion, and CO_2_ emissions [1,2]. The adoption of nature-based solutions, such as the use of cover crops for alleyways and under-vine floor management, can play a significant role in sustaining agroecosystem productivity and maintaining ecosystem services.

Cover crop cultivation in vineyards is a common practice worldwide and has various benefits. These include weed suppression and herbicide reduction [3], biological N fixation and nitrate (NO_3_^−^) leaching reduction [4,5,6], C sequestration, soil health improvement [7,8,9], biodiversity enhancement, and beneficial insect attraction [10]. Nutrient cycling improvement [11] and mitigation of the vine vigor [12] were also recognized. These expanded objectives underscore the evolving understanding and application of cover crop cultivation in vineyards.

The choice of plant species is a crucial factor that affects the effectiveness of cover cropping. The morphological and physiological attributes of cover crop species affect their role in the vineyard ecosystem. These attributes include the shoot and root morphology, the vegetative growth rate, the nutrient uptake and utilization efficiency, and the N and C contribution to the soil through biomass decay or incorporation [13]. Moreover, the root exudates also influence the soil biota and processes [14]. These characteristics determine the function of cover crop species in relation to grapevines and the soil environment [15]. Therefore, the choice of cover crop species should consider these aspects and their compatibility with the goal and the cropping system. An inappropriate choice of cover crop species can lead to undesirable outcomes, such as competition for water and nutrients with the grapevine and consequently reduction in yield and yield quality, as well as harboring insect pests and diseases [14].

Cover crops enhance C sequestration in vineyard ecosystems by transforming atmospheric CO_2_ into organic matter through photosynthesis [9]. This organic matter is then transferred to the soil via shoot and root decomposition, increasing the soil organic C stocks [16]. This process not only improves soil fertility but also mitigates climate change by lowering the atmospheric CO_2_ concentration [17]. The rate of C sequestration can be optimized by selecting a cover plant species with high biomass production [18]. Moreover, the C/N ratio of these residues influences the resident time of the input C in the soil [19]. Hence, choosing plant species with high biomass production and a high C/N ratio can alter the soil C balance in favor of C sequestration. However, species that exhibit excessive height, thereby interfering with the fruiting zone in wine grapes or those that generate substantial biomass demanding frequent mowing throughout the growing season, are deemed undesirable for vineyards.

The problem of NO_3_^−^ leaching is more pronounced in the initial years of vineyard establishment [20], when the root system of the vines is not fully developed and cannot absorb the available N efficiently. The use of cover crops, particularly in drive-row, can help to alleviate this concern by reducing the downward movement of NO_3_^−^ and retrieving NO_3_^−^ from deep soil layers [21]. However, the inclusion of under-vine cover crops in newly established vineyards should be done with care by selecting non-competitive species to eliminate the chance of competition. Moreover, the residues of cover crop roots and shoots can increase the soil organic matter content and provide a suitable environment for the growth of soil microorganisms [22]. These microorganisms can immobilize mineral N in their biomass when they have access to a labile C source; therefore, in the short term, the availability of mineral N under cover crop can be decreased by microbial immobilization. In the Mediterranean vineyard agroecosystem, it was observed that the microbial biomass N was typically 2–3 times higher in the Rye (*Secale cereale*) and Trios 102 (*Triticale × Triosecale*) cover crops compared to ‘Cultivation’ treatment during the winter and spring seasons [23].

With the ever-increasing demand for organic wine and enhanced adoption of organic practices in semi-arid vineyards, cover crops offer benefits like fixing atmospheric N and lessening the need for synthetic fertilizers. Research has shown that grape fertility, yield, Ravaz index, and total soluble solids are significantly higher in winter cover crops plus buried pruning residue in the unfertilized vines, thus showing the reliability of reducing N mineral fertilization and related risks of excess NO_3_^−^ in groundwater [6]. A systematic review by Abad, et al. [24] examined 14 articles and found that legumes as cover crops increased the total and mineral N content in the soil due to their ability to fix atmospheric N in symbiosis with Rhizobium bacteria in their roots. In contrast, grasses as cover crops decreased the total N content more than other families, because they scavenged N from the soil. Symbiotic N_2_ fixation measurements are difficult due to the process’s inherent complexity, including plant-bacteria interactions and environmental influences. Direct measurement methods are lacking, and existing techniques often rely on indirect indicators or have limitations [25].

Two sequential experiments were conducted in a greenhouse using loamy sand field soil. The aim was to explore the potential of 23 plant species to enhance soil organic N content and carbon sequestration for possible adoption in semi-arid irrigated vineyards. The study’s goals of enhancing soil health, C sequestration, and N management in vineyards through cover crops align with the United Nations Sustainable Development Goals (UN SDGs), specifically contributing to Goal 2 (Zero Hunger), Goal 13 (Climate Action), and Goal 15 (Life on Land).

## 2. Materials and Methods

A greenhouse study was conducted to evaluate the suitability of cover crop species for utilization in irrigated vineyards at the Summerland Research and Development Centre of Agriculture and Agri-Food Canada, Summerland, BC, Canada (latitude 49°33′59″ N, longitude 119°38′12″ W). The research was divided into two sequential experimental phases.

### 2.1. Experiment 1: Cover Crop Screening

Twenty-three cover crop species were chosen based on a comprehensive literature review and consultations with agricultural experts and wine grape growers (Table 1). Seeds were germinated in flat trays filled with 2 cm field soil for two weeks. After germination, seedlings were carefully transplanted into pots, each with a 30 cm diameter and 20 cm height. Pots were filled up to the 17 cm mark with Skaha sandy soil (Brunisoli in Canadian Soil Classification; 3.8 kg ± 0.1 pot^−1^) collected from the Summerland Research and Development Centre’s experimental farm. The physical and chemical characteristics of the soil are summarized in Table 2. The seeding rates were chosen based on the literature and were calculated based on the surface area of the pots.

Greenhouse temperatures were maintained at an average of 25 °C during the day and 20 °C at night, with a 14 h photoperiod. The average light intensity during the photoperiod was 550 µmol/m^2^/s. Soil moisture was maintained at approximately field capacity (average 20% volumetric soil water content) throughout the experiment by weighing weekly and watering as needed. Pots were fertilized for phosphorous (P) using triple super phosphate and for potassium (K) using potassium muriate, based on soil test recommendations. Weeds were removed from the pots by hand weekly. The experiment was organized as a completely randomized block design with four replications for each of the twenty-three cover crop species and one plant-free control.

Cover crops were harvested four months from the start of the experiment. Aboveground and belowground parts were separated. Roots were washed on top of a 1 mm sieve under gentle running tap water and dewatered using a salad spinner. The biomass samples were weighed and immediately dried at 60 °C until they reached a constant weight (on average 48 h). Oven-dried samples were weighed and ground with a Retsch mill followed by ball milling. Tissue C and N concentrations were measured using LECO 628 (LECO Corporation, St. Joseph, MI, USA). Cover crop C and N contents were calculated by multiplying dry biomass, and tissue C and N concentrations.

Soil organic C (after carbonate removal using HCl 1.0 N) and total N before planting were determined using a LECO 628 CHN analyzer (LECO Corporation, St. Joseph, MI, USA). Soil NO_3_^−^ and ammonium (NH_4_^+^) at harvest were extracted with 2 *M* KCl using a 1:5 soil/solution ratio and then analyzed colorimetrically on an AutoAnalyzer 3 Segmented Flow Analyzer (SEAL Analytical Inc., Mequon, WI, USA).

Nitrogen use efficiency was assessed using several indices:

N uptake efficiency (NU_p_E) represents the percentage of readily available N (RAN, mineral N pot^−1^) that was taken up by the cover crop [26]. RAN is defined as the readily available N in the absence of cover crop (control) at harvest. The NU_p_E was calculated as follows:

$${\mathrm{NU}}_{p}E=\frac{Plant\;total\;N\;content\;(g)}{RAN\;(g)}\times 100$$

where RAN and plant total N content are calculated based on gr pot^−1^ for each cover crop.

N utilization efficiency (NU_t_E) reflects the efficiency of converting absorbed N into plant biomass [26]. It was calculated as follows:

$${\mathrm{NU}}_{t}E=\frac{Plant\;dry\;biomass\;(g)}{Plant\;total\;N\;content\;(g)}$$

where plant dry biomass is the total aboveground + belowground dry biomass in grams and plant total N content is N content in plant tissue (aboveground + belowground) in grams.

Soil N use efficiency (NUE_soil_) indicates the overall efficiency of soil N in producing plant biomass [26]. It was calculated as follows:

$${\mathrm{NUE}}_{\mathrm{soil}}=\frac{Plant\;dry\;biomass\;(g)}{RAN\;(g)}$$

Plant-derived soil potentially available N (SPAN) is the amount of N that can become available to plants over time as a result of soil N mineralization acceleration by the plant. It can be calculated as follows:

$$\begin{array}{ll} SPAN=Plant & total\;N\;content-[RAN\\ & -\;Residual\;soil\;mineral\;N\;in\;present\;of\;cover\;crop\;at\;harvest] \end{array}$$

The readily available N reliance index (RANRI) is a proposed novel indicator, suggested by authors, that quantifies the extent to which a plant relies on RAN for its total N needs. The RANRI value represents the percentage of the plant’s total N that is sourced from RAN.

$$\mathrm{RANRI}=\frac{RAN-Residual\;soil\;mineral\;N\;in\;present\;of\;cover\;crop\;at\;harvest}{Plant\;total\;N\;content}\times 100$$

### 2.2. Experiment 2: Grapevine Response to Cover Crops

The effect of preceding cover crops on vine growth was assessed by drying, milling (<1 mm), and incorporating cover crop shoots and roots from Experiment 1 into the soil extracted from Experiment 1 pots before planting grapevines. Soil dry weight for each pot was measured at 3.6 kg ± 100g. Greenhouse conditions and soil moisture were maintained as described for Experiment 1. Pots were not fertilized in Experiment 2 since no nutrients were transported from the pots. Merlot cuttings were placed in perlite and kept moist for six months prior to the second experiment. The self-rooted Merlot vines were transferred to the pots and grown for 5 months. Similar to Experiment 1, Experiment 2 was set up as a complete randomized block design with 24 treatments and 4 replications. Weeds were removed by hand weekly as needed. At harvest, aboveground and belowground grapevine parts were separated. Roots were washed and biomass samples were dried and ground as described for cover crops. Grapevine tissue C and N concentrations were measured, and C and N contents were calculated as described above for cover crops. Soil organic C (after carbonate removal with HCl 1.0 N) and total N at harvest were determined using a LECO 628 CHN analyzer (LECO Corporation, St. Joseph, MI, USA).

### 2.3. Statistical Analysis

Data were analyzed using JMP software version 17.0.0 (SAS Institute, Inc., Cary, NC, USA). The normality of data distribution was tested using the Shapiro–Wilk test. When normality could not be assumed, data were log-transformed. Data were analyzed using a one-way analysis of variance (ANOVA) by considering cover crop species as fixed factors and replications as random factors. When a treatment’s effect on a parameter was significant, differences between treatment means were evaluated using the LSD test at a significant level of *p* < 0.05. Only significant differences at *p* < 0.05 are reported as decreased or increased in the Section 3.

## 3. Results

### 3.1. Shoot and Root Dry Weights and R/S Ratio

**Figure 1:**
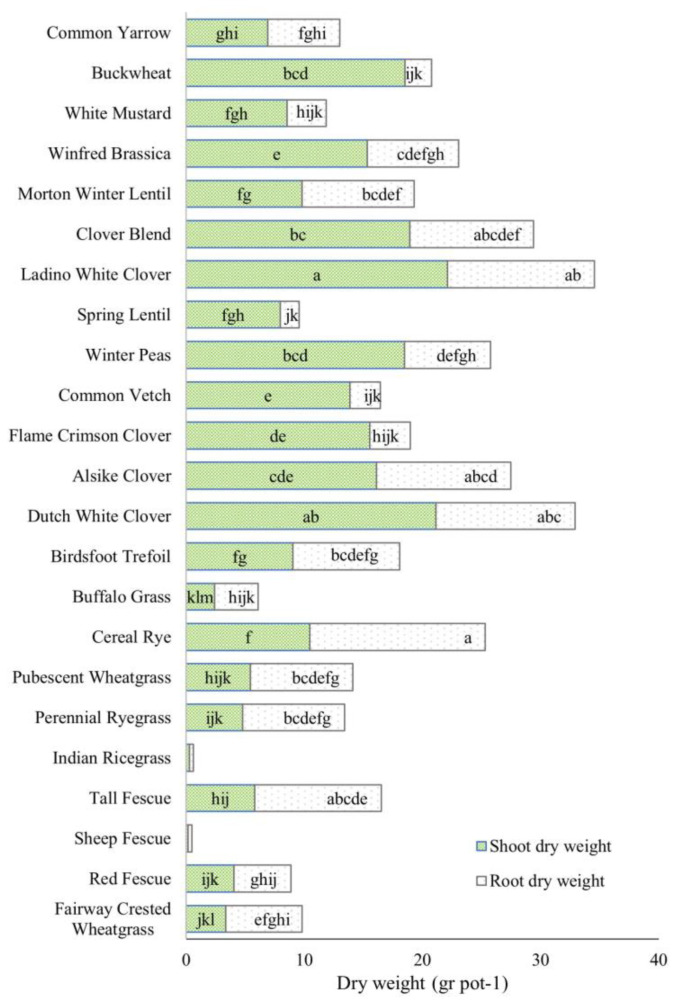
The shoot and root dry weights of 23 cover crop species. Each parameter followed by the same letter does not differ significantly (*p* ≤ 0.05) based on the LSD test.

The average shoot dry weight (SDW) of legumes was 15.3 g pot^−1^, while the average SDW of grasses was only 4.07 g (Figure 1). Among the legumes, Ladino white clover and Dutch white clover had the highest SDW, at 22.1 and 21.1 g, respectively. The grasses had the lowest SDW, except for fall rye, which had a moderate SDW of 10.5 g. Indian ricegrass and sheep fescue were poorly established and had the lowest SDW among all the species. Buckwheat, which belonged to the *Brassicaceae* family, had a high SDW of 18.5 g, which was greater than the average for legumes.

The root dry weight (RDW) of the plant species was significantly different (Figure 1). The RDW was greater in the legumes (7.94 g) than in the grasses (6.50 g), but the magnitude of the difference was smaller than in the SDW. The highest RDW was observed in fall rye (14.8 g), followed by Ladino white clover (12.4 g) and Dutch white clover (11.8 g).

In the comparative analysis of the root–shoot ratio (R/S ratio) among grasses, legumes, and forbs, no significant difference was observed within the grasses (Table 3). However, the mean R/S ratio for grasses, quantified as 1.57, was statistically higher than that of legumes (0.53) and forbs (0.47). Among the species studied, fairway crested wheatgrass and tall fescue exhibited the highest R/S ratios, with values of 1.95 and 1.84, respectively. Conversely, buckwheat (0.12), common vetch (0.19), spring lentil (0.20), and flame crimson clover (0.22) demonstrated the lowest R/S ratios.

### 3.2. Total Dry Weight (TDW), Total C Content (TCC), and Total N Content (TNC)

The mean values of TDW, TCC, and TNC for each plant species are shown in Table 3. The highest TDW values were observed for Ladino white clover (34.5 g pot^−1^), Dutch white clover (32.9 g pot^−1^), and clover blend (29.4 g pot^−1^). These three species are significantly different from all the other species, except alsike clover (27.4 g pot^−1^). The lowest dry-weight producers are fairway crested wheatgrass (9.8 g pot^−1^), spring lentil (9.5 g pot^−1^), red fescue (8.9 g pot^−1^), and buffalo grass (6.0 g pot^−1^). These four species are significantly different from all the other species. Linear regression analysis was used to investigate the relationship between TDW, TCC, and TNC of plant species. The results showed that TCC and TNC increased linearly with TDW, with high coefficients of determination (R^2^) of 0.97 and 0.94, respectively.

To gain clearer insights into the levels of C and N sequestration and their potential impact on the vineyard ecosystem, the plant species were grouped into four categories based on their TDW, TCC, and TNC contents:

Group 1: High TDW, TCC, and TNC. The plants in this group include Ladino white clover, Dutch white clover, and clover blend.

Group 2: High TDW and TCC, moderate TNC. The plants in this group are fall rye, winter peas, alsike clover, and Winfred brassica.

Group 3: Moderate TDW, TCC, and TNC. The plants in this group are birdsfoot trefoil, flame crimson clover, common vetch, buckwheat, Morton winter lentil, tall fescue, pubescent wheatgrass, and perennial ryegrass.

Group 4: Low TDW, TCC, and TNC. The plants in this group are fairway c. wheatgrass, red fescue, buffalo grass, white mustard, common yarrow, and spring lentil.

### 3.3. Shoot C and N Concentrations and C/N Ratio

The analysis revealed a significant effect (*p* < 0.01) of plant species on the shoot C concentration (SCC) (Table 3). However, this effect was not evident when comparing the mean SCC values across 18 of the 23 plant species, which ranged from 38.0% to 41.5% (LSD_0.05_ = 4.042). Only three species—red fescue (36.1), common yarrow (35.8), and perennial ryegrass (34.9)—exhibited values that fell below 38%. Additionally, no specific pattern was observed in terms of SCC among plant families.

The shoot N concentration (SNC) varied significantly among the plant species (Table 3). The legume species had the highest mean SNC of 2.50%, which was significantly higher than the mean SNC of the grasses (1.49%) and forbs (1.29%). Within the legumes, clover blend and alsike clover had the highest SNC, with 2.96% and 2.94%, respectively, followed by winter peas (2.84%) and Ladino white clover (2.68%). The lowest SNC was observed in Winfred brassica (0.88%) and buckwheat (0.89%), which belonged to the Brassicaceae and Polygonaceae families, respectively. Among the grass species, perennial ryegrass and red fescue had the highest SNC, with 1.9% and 1.83%, respectively.

The shoot C/N ratio was strongly influenced by the SNC, as shown by the similar pattern of variation among the plant species (Table 3). A correlation analysis was performed to examine the relationship between the shoot C/N ratio and the shoot N and C contents. The results indicated that there was a significant and negative polynomial correlation (R^2^ = 0.95) between the shoot C/N ratio and the SNC. However, there was no significant correlation between the shoot C/N ratio and the SCC.

### 3.4. Root C and N Concentrations and C/N Ratio

The root C concentration (RCC) did not show any significant difference among the plant species (Table 3). The mean RCC ranged from 20.01% to 37.39%, with a standard deviation of 9.61%. The high standard deviation indicates that there was a large variation in the RCC within each plant species, which might have masked the potential effect of the plant species’ identity on the root C content.

The plant species from the legumes, except for birdsfoot trefoil and Morton winter lentil, had a significantly higher root N concentration (RNC, 1.82%) than the plant species from the other families (0.89%). In legumes, winter peas had the highest RNC (2.81%), while birdsfoot trefoil had the lowest RNC (1.17%). Among the grasses and forbs, there were no statistically significant differences in the RNC (Table 3). The highest RNC among the grasses and forbs species was observed in red fescue (1.13%), while the lowest RNC was observed in Winfred brassica (0.73%).

The root C/N ratio differed significantly among the plant species (Table 3). The plant species from the legumes had a significantly lower root C/N ratio (18.3) than the plant species from the other families (27.7). Winter peas had the lowest root C/N ratio (11.4), while common yarrow had the highest root C/N ratio (39.1). However, there was no significant correlation between the root C/N ratio and the RCC or RNC.

### 3.5. Soil Residual NH_4_^+^, NO_3_^−^, and Mineral N

Soil residual NH_4_^+^ concentration varied significantly among the plant species, but not between most of the pairwise comparisons (Table 4). The highest residual NH_4_^+^ levels were observed in the control treatment and in the plots with sheep fescue and Indian ricegrass, which had the lowest plant establishment rates. Common vetch had the highest soil residual NH_4_^+^ concentration among the species and was significantly different from most of them.

Soil residual NO_3_^−^ concentrations also differed significantly among the plant species, but it was not affected by the plant’s functional group (Table 4). The control treatment and the pots with sheep fescue and Indian ricegrass had the highest residual NO_3_^−^ levels, which corresponded to their lowest plant establishment rates. The lowest residual NO_3_^−^ levels were found in the pots with tall fescue and clover blend, but they were not significantly different from most of the other species. Residual mineral N followed a similar pattern as residual NO_3_^−^. Additionally, there were no significant changes in the residual NH_4_^+^/NO_3_^−^ ratio.

### 3.6. RAN, SPAN, and RANRI

**Figure 2:**
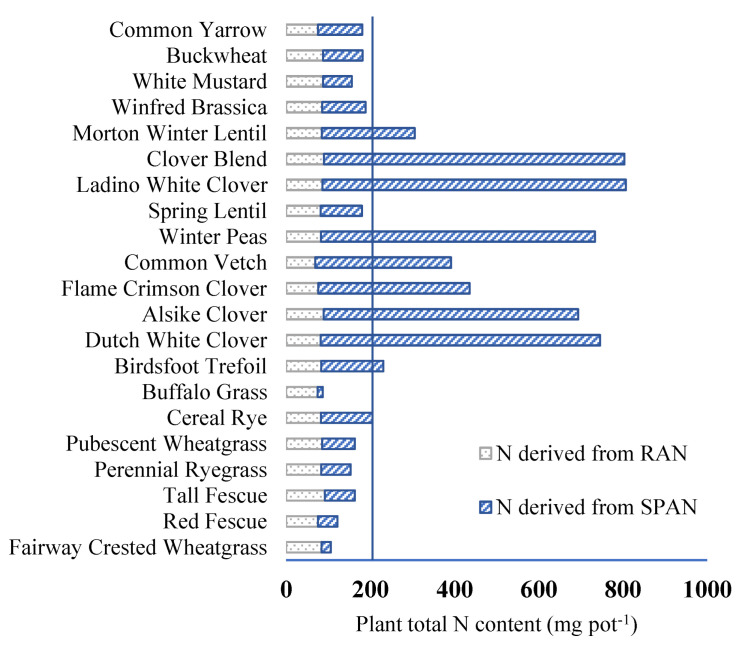
The total N content of cover crop species is divided into two parts: (i) plant N derived from RAN, which is calculated as soil mineral N in the absence of cover crop (control) at harvest minus residual soil mineral N in the presence of cover crop at harvest, and (ii) plant N derived from plant-derived soil potentially available N (SPAN). The vertical line shows the highest amount (205 mg pot^−1^) of TNC in non-legumes.

Legumes generally demonstrated the greatest reduction in RAN, followed by forbs and grasses with comparable levels (Figure 2). Grass species demonstrated a considerable range in the reduction of RAN, with tall fescue exhibiting the maximum reduction (91 mg pot^−1^) and red fescue the minimum reduction (77 mg pot^−1^). Forb species reduced RAN at levels comparable to grasses, with white mustard and buckwheat displaying the highest reductions (87 mg pot^−1^) and common yarrow displaying the lowest reduction (76 mg pot^−1^).

The results demonstrated a significant disparity in SPAN among the plant groups, in contrast to the lack of significant differences observed in residual N. Grasses exhibited the lowest SPAN (60 mg pot^−1^), followed by forbs (93 mg pot^−1^), with legumes possessing the greatest levels (450 mg pot^−1^). Within these groups, fall rye (122 mg pot^−1^), Ladino white clover (720 mg pot^−1^), and common yarrow (105 mg pot^−1^) represented the highest SPAN levels in their respective categories. Figure 2 includes a vertical threshold line (205 mg pot^−1^) indicating the TNC observed in non-leguminous species. Notably, only a single legume, spring lentil, fell below this threshold. The negligible residual N levels in most species suggest extensive uptake of both RAN and mineralized N.

Grasses had the highest RANRI, ranging from 40.4% (fall rye) to 85.5% (buffalo grass). Forbs displayed intermediate RANRI values between 41.8% (common yarrow) and 56% (white mustard). RANRI values were significantly lower in legumes, ranging from 10.7% (Ladino white clover) to 45.7% (spring lentil).

### 3.7. N Use Efficiency

NUE was calculated based on three indicators: N uptake efficiency (NUpE), N utilization efficiency (NUtE), and soil N efficiency ratio (NUE_soil_). The cover crop species varied in their NUE indicators, depending on their biomass production, N uptake, and N content (Table 4). The highest NUtE values were observed for fall rye (123.3 g g^−1^), Winfred brassica (127 g g^−1^), and buckwheat (114 g g^−1^). Forbs exhibited the highest NUtE (96.9), followed by grasses (91.1), with legumes demonstrating the lowest value (48.5). The highest NUpE values were observed for Ladino white clover (85.2%), clover blend (84.9%), and Dutch white clover (78.8%). NUpE values for grasses (15.1%), legumes (56.3%), and forbs (22.0%) were significantly different. The highest NUE_soil_ values were observed for Dutch white clover (347 g g^−1^), Ladino white clover (365 g g^−1^), and clover blend (310 g g^−1^). Legumes exhibited the highest NUE_soil_ (245), followed by forbs (183) and grasses (142).

### 3.8. Grapevine Growth and N Concentration

Cover cropping significantly altered the grapevine shoot N concentration (GSNC) and the C/N ratio (GSC/N). Multiple regression analysis showed that the cover crop’s TDW, soil NO_3_^−^, and C/N ratio explained 51.2% of the variation in GSNC. The GSNC positively correlated with soil NO_3_^−^ (R^2^ = 0.31) and cover crop C/N ratio (R^2^ = 0.17) but negatively correlated with the cover crop’s TDW (R^2^ = 0.31). Species such as clover blend, tall fescue, and flame crimson clover, which had the highest NO_3_^−^ uptake (lowest residual NO_3_^−^), resulted in the lowest concentrations of N in grape seedlings (Table 5).

While cover crop species did not significantly differ in their influence on leaf number and lower and upper leaves SPAD values, the variations observed between cover crop species in these parameters followed a trend similar to that of GSNC (Table 5). Chlorosis in grape leaves led to a negative correlation (R^2^ = 0.34) between grapevine shoot dry matter and GSNC, obscuring the relationship between GSDM and cover crop species. The high variability in GRFM values masked any potentially significant relationship between this attribute and other parameters.

## 4. Discussion

### 4.1. Interpretation of Cover Crop Shoot and Root Biomass Balance

The study results indicated that the tested cover crop species exhibited significant differences in SDW, RDW, and R/S ratio. These differences underscore distinct growth strategies and adaptive mechanisms among the species. The legumes, especially clovers, buckwheat, and Winfred brassica, had the greatest SDW, indicating that they invested more resources in their above-ground biomass than the other species (Figure 1). Legumes displayed an average SDW of 15.3 g pot^−1^, while forbs had 12.3 g pot^−1^, and grasses had 4.07 g pot^−1^ (Figure 1). Cover crops that exhibit high SDW offer potential management and environmental benefits. Research supports their role in enhancing soil organic matter and C sequestration [9], mitigating soil erosion and runoff [8], and promoting soil microbial activity and diversity [23,27]. Additionally, rapid establishment and soil coverage by these cover crops can suppress weed growth, potentially reducing reliance on herbicides [3,28].

However, in regions with limited water availability or soil fertility, selecting species exhibiting moderate SDW, such as Fall rye, Common Vetch, and Morton winter lentil, may be a more suitable strategy. Grasses often demonstrate greater tolerance to water scarcity and can thrive in less fertile soils [29,30].

In the context of SDW, legumes exhibited a clear dominance (Figure 1). However, this distinction narrowed considerably when considering RDW. Fall rye and tall fescue ranked first and fifth, respectively, in terms of RDW production. Ladino white clover, Dutch white clover, and alsike clover were interspersed between these two species. The production of high RDW carries several benefits that contribute to the overall health and stability of the ecosystem.

One of the primary advantages of high RDW is the enhancement of aggregate formation and stability [31,32]. The extensive root systems of these plants enhance soil aggregation, thereby reducing the risk of soil displacement and erosion [33]. This characteristic is particularly beneficial for vineyards, which are predominantly located on slopes [34]. Additionally, legume species with coarse root axes generate a greater number of macropores compared to species with fine roots [35]. This difference in root structure results in increased soil hydraulic conductivity and a reduction in surface runoff [33,36]. In addition, cover crops with fine-branched root systems (e.g., ryegrass, rye) are demonstrably more effective in preventing concentrated flow erosion compared to those with thick roots (e.g., white mustard, fodder radish) [37]. High RDW not only promotes plant growth but also stimulates soil microbial activity through increased root exudates [14,38], thereby enhancing C and N cycling within the soil ecosystem [39].

The R/S ratio is a key indicator of plant growth and resource allocation. Plants adjust their R/S biomass ratio as a strategy to adapt to environments with limited resources [40]. We found that grasses had a significantly higher mean R/S ratio than other plant groups (Table 3). Evidence suggests that legumes dynamically allocate resources between root growth and N fixation. When soil N levels are sufficient, reduced investment in root development may be observed due to the high energetic cost of symbiotic N fixation [41]. The R/S ratio was influenced more by the RDW than by the SDW, suggesting that the root biomass was more variable and responsive to species differences than the shoot biomass. Buckwheat exhibited the lowest R/S ratio, recorded at 0.12. This observation is attributed primarily to its significantly reduced RDW, which was measured at 2.22 g pot^−1^, in contrast to its substantial SDW. The underlying cause of this disparity may be linked to Buckwheat’s inherent drought avoidance characteristics, as suggested by Selwal, et al. [42]. Consequently, the R/S ratio emerges as a potential metric for assessing drought resilience, as supported by the findings of Mathew, et al. [43] and Pirnajmedin, et al. [44]. These metric gains are particularly important when selecting appropriate cover crops for semi-arid regions like the Okanagan Valley in Canada, where drought tolerance is a critical factor.

A high R/S ratio in cover crops may have some advantages, such as high efficiency in the absorption of mineral elements and N scavenging in deep soil. These benefits may be more pronounced in grass species that exhibit a lower base temperature compared to grapevines, thereby enabling their growth during autumn, subsequent to the cessation of grapevine growth and prior to the initiation of grapevine growth in spring. This capability of grasses to enhance soil properties [45] and water retention [46] while simultaneously minimizing competition with grapevines underscores their potential utility in viticulture.

In contrast, Morlat and Jacquet [47] observed that competition between vines and cover crop species reduced the density of vine roots in the inter-row while promoting deeper root development within the vine row. Klodd, et al. [48] indicated that grapevines adjust their root systems to compete with alleyway cover crops, particularly affecting phosphorus uptake, while the impact on water and N is less significant. In contrast, Sweet and Schreiner [49] suggested that competition between grass cover crops and grapevines might be more related to N than water availability. This is because grapevines can compensate for water competition by extending their roots deeper into the soil, but N availability is typically lower in these deeper layers. The extent of this competition and resource limitation varies with the environmental conditions.

Similarly, a low R/S ratio in cover crops can offer certain advantages, including increased aboveground biomass production and enhanced weed suppression. A low R/S ratio in cover crops, however, can have disadvantages, such as increasing water and nutrient demands [50]. These sensitivities may be unsuitable for vineyard production environments characterized by low-fertility soils and limited moisture availability [51]. Therefore, a low or high R/S ratio may be beneficial or detrimental to the vineyard, depending on the specific conditions and management practices, such as variety vigor or environmental conditions [51]. A potential resolution to this issue could be the strategic utilization of cover crops. Specifically, cover crops with a low R/S ratio could be employed for the under-vine areas, while those with a high R/S ratio could be used for the alley cover crops. This approach could potentially optimize resource competition and allocation between the grapevines and the cover crops. However, further research is needed to validate this hypothesis and to explore its practical implications in viticulture.

### 4.2. Analysis of Cover Crops Total Biomass, C, and N Allocation

The findings presented in the study provide valuable insights into the TDW, TCC, and TNC of various plant species (Table 3). Based on the range of these parameters and literature, we categorized the plant species into four groups, offering a comprehensive understanding of their growth characteristics and potential ecological significance.

Group 1, consisting of cover crop species, demonstrated high TDW, TCC, and TNC values. This suggests that these species are highly productive and could play a significant role in C and N cycling in the ecosystem. Their high biomass production characteristics could also contribute to soil organic matter and improve soil fertility. Therefore, they could be suitable candidates for cover crops in vineyards, both in alleyways and under-vine. However, blending them with grasses is recommended for alleyways [52]. The study conducted by Guzmán, et al. [32] indicated that the spontaneous cover crop with low biomass production had only a moderate or negligible impact on soil properties when compared to the bare soil vineyards. Contrastingly, the most effective strategy appeared to be cultivating vineyards with spontaneous cover crops that could achieve a high biomass production, exceeding 0.91 Mg ha^−1^ per year [32].

Group 2, which includes cover crop species, showed high TDW and TCC, but moderate TNC values. This indicates that while these species are effective in sequestering C, their contribution to N cycling may be less pronounced. Novara, et al. [2] observed a 6% increase in SOC in flat areas and a 9% increase in sloping vineyards following five years of cover crop management. Fageria, et al. [29] reported that rye (*Secale cereale* L.) could accumulate up to 100 kg N ha^−1^. Therefore, they can be considered suitable cover crop candidates for vineyard alleyways.

Group 3, which includes cover crop species, showed moderate TDW, TCC, and TNC values. These species strike a balance between biomass production and resource use efficiency, making them ideal for environments with limited resource availability. Consequently, they could be considered good candidates for under-vine cover cropping.

Lastly, Group 4, which includes cover crop species, showed moderate TDW, TCC, and TNC values. These species might be adapted to low-resource environments and could be useful in conservation and restoration projects where the goal is to establish vegetation with minimal inputs.

The linear relationship observed between TDW, TCC, and TNC highlights the interconnectedness of these parameters and suggests that increasing plant biomass could simultaneously enhance C and N sequestration. This finding is pivotal for selecting appropriate cover crop species, where biomass production is a key criterion. However, considering potential interference is crucial, particularly in under-vine cover cropping. High biomass production could intensify resource utilization and competition between cover crops and grapevines [39,47,53]. Additionally, some cover crops may exhibit allelopathic effects on grapevines [12,21,28,54], while others might pose challenges due to their climbing growth habit [55]. While these challenges exist in cultivating under-vine cover crops, their implementation in alleyways offers the potential for effective weed and disease management [14,21]. With increasing public pressure on policymakers to ban herbicides, particularly glyphosate, in vineyards, cultivation and cover cropping remain the only two viable options for under-vine weed control. Among these, under-vine cover crops are preferable due to their numerous potential benefits, including improved soil health, enhanced biodiversity, and reduced erosion.

### 4.3. Implications of Cover Crop Shoot and Root C and N Dynamics

Although SNC varied significantly among different plant species, SCC remained consistent across species, ranging from 38 to 41.5% (Table 3). This indicates that the C content of plant biomass was not influenced by species type. Legume species had the highest SNC values, ranging from 1.95% (Morton winter lentil) to 2.96% (Clover Blend). This is consistent with previous studies that reported higher SNC values for legumes than for grasses and forbs [4,56]. On the other hand, fast-growing crops like Winfred Brassica, Buckwheat, and Fall rye, known for their high biomass production, exhibited the lowest SNC values. The SNC and the C/N ratio of plant biomass significantly impact soil C and N cycling [57]. High SNC and low C/N ratios suggest a greater potential for N release into the soil through mineralization, enhancing soil fertility and N availability for subsequent crops [14]. This results in shorter C resident times in the soil, particularly in the lighter soil textures common in vineyards [57]. Conversely, low SNC and high C/N ratios indicate that plant biomass can effectively sequester C in the soil, as slower decomposition rates allow more time for the establishment of soil’s physical and chemical protections, making C more likely to be stored for a longer period of time [14,57]. Therefore, selecting plant species for cover cropping should be guided by management objectives and the trade-offs between C and CN cycling.

The results showed that root C and N concentrations and their C/N ratio had a similar pattern to shoots in different plant species (Table 3). This suggests that there is a coordination between roots and shoots in terms of C and N allocation and use efficiency. However, the root data had high variability, likely due to the unpredictable nature of root growth and the inherent inaccuracy associated with root quantification. This limitation should be addressed in future studies by using more standardized and precise methods of root sampling and analysis. Legumes exhibit higher RCC than other plant species. This characteristic could partially compensate for their lower shoot C, as root-derived C contributes more significantly to stable soil C pools than C derived from above-ground plant residues [58]. The study by Rasse, et al. [59] demonstrated that root-derived C persists in soils an average of 2.4 times longer than C originating from above-ground plant residues.

### 4.4. Residual Soil N Dynamics after Cover Crop Harvest

This study explored the intricate relationship between cover crop species and soil N dynamics. The most surprising result was the observed negative correlation between dry matter production and residual N across all plant species employed. Notably, even legume varieties, renowned for their N-fixing prowess, were unable to impede this soil N depletion (Table 4). Two outliers emerged from this overarching trend: tall fescue exhibited a more pronounced reduction in soil mineral N than anticipated, while common vetch displayed a comparatively diminished impact.

Within the constraints of this study, each pot contained a finite volume of soil, thereby limiting the quantity of RAN to approximately 102 mg pot^−1^. Intriguingly, the N content within the entirety of the plant exceeded the RAN, with the exception of three species characterized by low TDW. Irrespective of the potential N losses, it was postulated that the surplus N could be attributed to either mineralization processes or biological N fixation. In species known for biological N fixation, distinguishing between these two N sources proves challenging; accordingly, we designated this category as SPAN.

The observed five-fold higher SPAN in Ladino white clover compared to fall rye can be attributed to biological N fixation. Even when legumes were excluded, substantial SPAN levels were observed in several species, exceeding RAN levels in some cases. This phenomenon can be interpreted in light of the documented variations in mineralization rates attributable to different plant species [60,61]. It is crucial to acknowledge that plants situated in N-deficient environments seem to significantly utilize organic N as a source [62].

To quantify the contribution of RAN to TNC, the RANRI metric was employed. RANRI values exhibited variation across functional groups, with grasses averaging 61%, forbs at 47%, and legumes at a significantly lower 20%. This translates to an estimated 39%, 53%, and 80% of TNC in grasses, forbs, and legumes, respectively, originating from SPAN. Intriguingly, seven legume species displayed a particularly low RANRI range (11–17%), implying a substantial influx of N (83–90%) through a pathway independent of RAN. This observation, alongside the absence of a similar trend in other functional groups, strongly suggests biological N fixation BNF as a potential mechanism for this unaccounted-for N acquisition. Supporting this hypothesis, Pirhofer-Walzl, et al. [56] reported that legumes can derive 75–95% of their N needs through symbiotic N_2_ fixation.

### 4.5. Efficiency of Cover Crop N Uptake and Utilization

NUE was assessed using three distinct metrics: NU_p_E, NU_t_E, and NUE_soil_. Forbs demonstrated the highest NU_t_E (96.9), followed by grasses (91.1), with legumes exhibiting the lowest value (48.5). NU_t_E is a valuable metric for identifying plants with superior capacity to generate yield proportional to plant tissue N content, making it particularly suitable compared to grasses or forbs. Schulte auf’m Erley, et al. [63] observed a negative correlation between N application rates and NU_t_E, suggesting that the diminished NU_t_E in leguminous plants may be an evolved mechanism to intensify biological N fixation processes.

NU_p_E values showed significant variation across grasses (15.1%), legumes (56.3%), and forbs (22.0%). NU_p_E quantifies plant assimilation of soil N. Observations indicate that variations in RAN concentrations are not significantly correlated with plant functional groups (Table 4). Consequently, the elevated NU_p_E observed in legume species is likely a result of their intrinsic biological N fixation processes. This hypothesis is corroborated by the RANRI index. There is a positive correlation between TDW and NU_p_E, while a negative correlation exists with NU_t_E. These findings are consistent with the results presented by Williams, et al. [64]. Low NU_p_E indicates that N remaining in the soil after harvest or unused by the plant at the end of the season is susceptible to loss [26].

Legumes displayed the highest NUE_soil_ (245), trailed by forbs (183), and grasses (142). NUE_soil_ serves as a robust indicator of a crop’s potential to generate substantial biomass per unit of available N. Given the observed low NU_t_E in legumes, it can be inferred that a high NUE_soil_ could potentially indicate biological N fixation. This inference is further substantiated by the negative correlation identified between NUE_soil_ and RNARI.

Intercropping legume cover crops with species exhibiting high NU_t_E has the potential to intensify N and C sequestration. This synergistic effect could arise from the transfer of N fixed by legumes to companion plants, leading to increased biomass production with a higher C/N ratio.

### 4.6. Grapevine Growth and N Status

This study highlights the significant impact of previous cover crop management on GSNC and the GSC/N ratio (Table 5). Our multiple regression analysis revealed that cover crop TDW, residual soil NO_3_^−^, and cover crop C/N ratio collectively explain over 50% of the variation in GSNC. The positive correlation between GSNC and residual soil NO_3_^−^ (R^2^ = 0.31) suggests that cover crops influencing soil N availability can subsequently impact N uptake by grape seedlings. Cover crop species, including Flame Crimson Clover, Clover Blend, and Tall Fescue, exhibited robust NO_3_^−^ uptake capabilities. This suggests a potential role in transient soil N immobilization, likely contributing to reduced N availability prior to grape seedling establishment. Finney, et al. [30] found that while higher cover crop biomass offered advantages like weed control, reduced NO_3_^−^ leaching, and increased biomass N, it also led to lower N availability and negatively affected subsequent corn yields. Similarly in vineyard study, Celette and Gary [65] found that implementing a permanent grass cover crop in a water-limited environment led to both water and N stress in grapevines compared to bare soil. However, it is important to note that this experiment simulates an under-vine environment and its findings may not directly translate to alleyway cover cropping scenarios [12]. Pérez-Álvarez, et al. [66] demonstrated that a barley cover crop reduced soil N availability from the first year, leading to decreased leaf N and vine vigor by the third year. Conversely, a clover cover crop increased soil N availability from the second year, resulting in elevated leaf N content in the third and fourth years.

The observed negative correlation between GSNC and cover crop TDW (R^2^ = 0.31) suggests an indirect relationship. While strategies to enhance cover crop TDW may contribute to NO_3_^−^ reduction, this appears to be inversely correlated with GSNC. This observation aligns with the understanding that increased biomass production in cover crops or green manures can lead to a reduction in available soil mineral elements [65].

The positive correlation between GSNC and cover crop C/N ratio (R^2^ = 0.17) suggests that the rate of cover crop residue decomposition may also play a role in N dynamics. Cover crops with a wider C/N ratio tend to decompose slower [57], which could prolong the period of N immobilization, negatively affecting N availability for grape seedlings in the short term [36].

While the existing literature predominantly indicates a negative correlation between the C/N ratio and subsequent plant N accessibility [67,68,69], this study presents an unexpected positive relationship between the C/N ratio and GSNC. Despite the incorporation of low C/N ratio biomass from legume cover crops, the grape seedlings exhibited N deficiency, likely due to insufficient time for significant mineralization of plant residues to offset NO_3_^−^ depletion caused by the cover crops; this statement is supported by [66]. The rate of N derived from the decomposition of grass and legume litter within a short timeframe (8–20 weeks) has been reported to be less than 2% [70,71]. While the decrease in mineral N potentially stimulated gross mineralization, it is likely that this led to an overall decline in the net mineralization rate. This aligns with the findings of Mooshammer, et al. [72], who demonstrated that under N-limited conditions, microbes exhibit high N use efficiency by retaining fixed organic N, resulting in reduced N mineralization. While incorporating cover crops is essential for long-term soil health and fertility, their potential short-term effects on N availability warrant careful consideration, particularly when utilized as an under-vine cover crop during the critical establishment phase of grapevines.

It is crucial to acknowledge that this experiment employed a sequential design where cover crops were established prior to grape seedling cultivation. This allowed cover crops ample time to deplete soil N, creating an environment where grape seedlings faced reduced N availability. While this scenario deviates from typical vineyard management practices, the experiment’s objective was to assess the potential for N release from cover crops to the primary crop during concurrent growth. The results suggest that such immediate release may not be substantial. It is essential to note that in natural settings, plant residues typically decompose over a more extended period, potentially altering N dynamics. This experiment indicates that, for optimal results, vigorous cover crops should not be planted directly under vines and that adequate spatial separation is crucial during the establishment phase. Furthermore, it highlights the need for additional research to investigate the temporal patterns of nutrient release, residue decomposition rates, and soil microbial community responses.

## 5. Conclusions

This study highlights the potential of various cover crop species to enhance C sequestration and soil N dynamics in vineyard ecosystems. Among the established cover crops, the C sequestration potential ranged from 0.267 to 1.69 Mg ha^−1^, and the N input potential ranged from 12.3 to 114 kg ha^−1^ in four months. Legume species, particularly Ladino white clover, Dutch white clover, and Clover blend, showed superior biomass production and N efficiency compared to grasses and forbs. These legumes demonstrated higher N uptake and utilization efficiencies, enhancing soil N storage. The Readily Available N (RAN) Reliance Index (RANRI) is introduced as a novel indicator for quantifying the extent to which a plant relies on RAN for its total N requirement. Intriguingly, legume species displayed a particularly low RANRI range (11–17%), implying a substantial influx of N (83-90%) through a pathway independent of RAN. This capability of legumes is crucial for promoting sustainable practices and improving the ecological footprint of agricultural systems, particularly vineyards.

Cover crops significantly influenced grapevine N concentration and growth, with species such as Clover blend and tall fescue efficiently absorbing soil NO_3_^−^ and reducing N uptake in grape seedlings. Therefore, close attention to grapevine N demand is required, even with legume cover crops. Next steps include field trials to assess cover crop performance under natural vineyard conditions, determining key cover crop selection criteria, and investigating nutrient release and decomposition patterns. Synchrony between cover crop species and grapevine water demand is crucial for successful cover cropping in irrigated vineyards.

This study demonstrates significant benefits applicable to other agricultural systems. The superior cover crop species introduced in this study have the potential to improve water retention, enhance nitrogen retention, and reduce nitrogen leaching, thereby protecting resources. They also increase carbon sequestration, improving soil health and mitigating climate change. For growers, cover crops lead to higher yields per input due to enhanced soil fertility and a reduced need for synthetic fertilizers. Additionally, they suppress weeds, decreasing reliance on herbicides and reducing the number of tractor passes, which lowers fuel consumption and operational costs. These advantages make selecting cover crops properly a sustainable and economically beneficial practice for diverse cropping systems.

## Figures and Tables

**Table 1:** Common and scientific names and functional types of 23 cover crop species used in the experiment.

Common Name	Scientific Name	Functional Family/Type
Fairway crested wheatgrass	*Agropyron cristatum*	Poaceae/Grass
Red fescue	*Festuca rubra*	Poaceae/Grass
Sheep fescue	*Festuca ovina*	Poaceae/Grass
Tall fescue	*Festuca arundinacea*	Poaceae/Grass
Indian ricegrass	*Oryzopsis hymenoides*	Poaceae/Grass
Perennial ryegrass	*Lolium perenne*	Poaceae/Grass
Pubescent wheatgrass	*Thinopyrum intermedium*	Poaceae/Grass
Fall rye	*Secale cereale*	Poaceae/Grass
Buffalo grass	*Bouteloua dactyloides*	Poaceae/Grass
Birdsfoot trefoil	*Lotus cornicalatus*	Fabaceae/Legume
Dutch white clover	*Trifolium repens cv. Dutch*	Fabaceae/Legume
Alsike clover	*Trifolium hybridum*	Fabaceae/Legume
Flame crimson clover	*Trifolium incarnatum*	Fabaceae/Legume
Common vetch	*Vicia sativa*	Fabaceae/Legume
Winter peas	*Pisum sativum*	Fabaceae/Legume
Spring lentil	*Lens culinaris cv. Spring*	Fabaceae/Legume
Ladino white clover	*Trifolium repens cv. Ladino*	Fabaceae/Legume
Clover blend (Ladino white clover, alsike clover, sweet clover, double-cut red clover, crimson clover)	…, *Melilotus officinalis*, *Trifolium pratense*	Fabaceae/Legume
Morton winter lentil	*Lens culinaris cv. Morton Winter*	Fabaceae/Legume
Winfred brassica	*Brassica napus cv. Winfred*	Brassicaceae/Forbs
White mustard	*Sinapis alba*	Brassicaceae/Forbs
Buckwheat	*Fagopyrum esculentum*	Brassicaceae/Forbs
Common yarrow	*Achillea millefolium*	Asteraceae/Forbs

**Table 2:** Mean soil physical and chemical properties (n = 3).

Soil Organic C	Total N	C/N Ratio	EC ^2^	pH	Clay	Silt	Sand	Texture ^3^
%	%		dS m^−1^	CaCl ^1^	%			
1.51	0.148	10.26	0.41	6.94	13.36	38.11	48.52	loam

^1^ Soil pH measured in a CaCl_2_ solution (soil/solution ratio of 1:2, m/v). ^2^ EC: electrical conductivity. ^3^ Soil texture were measured using an automated soil particle-size analyzer.

**Table 3:** Characterization of various cover crop species on the root/shoot ratio (R/S), total dry matter (TDW), total C content (TCC), total N content (TNC), shoot C, N and C/N ratio, root C, N and C/N ratio.

Plant Species	R/S	TDW (g pot^−1^)	TCC (g pot^−1^)	TNC (g pot^−1^)	Shoot			Root		
					C (%)	N (%)	C/N Ratio	C (%)	N (%)	C/N Ratio
Fairway C. wheatgrass	1.95	9.8	2.58	0.106	41.5	1.68	24.7	21.4	0.86	23.8
Red fescue	1.19	8.9	2.71	0.122	36.1	1.83	20.1	27.5	1.13	23.9
Sheep fescue	1.63	0.47	--	--	--	--	--	--	--	--
Tall fescue	1.84	16.5	5.11	0.163	39.6	1.21	33.3	27.2	0.90	29.7
Indian ricegrass	1.15	0.63	--	--	--	--	--	--	--	--
Perennial ryegrass	1.81	13.6	3.35	0.153	34.9	1.90	18.4	22.0	0.76	27.3
Pubescent wheatgrass	1.60	14.1	5.14	0.163	39.6	1.44	27.5	34.3	0.97	35.1
Fall rye	1.42	25.3	7.21	0.205	39.0	0.95	41.2	22.5	0.76	29.4
Buffalo grass	1.53	6.0	1.89	0.087	39.2	1.40	28.7	25.6	1.04	23.2
Birdsfoot trefoil	1.00	18.0	6.52	0.231	39.9	2.15	19.1	33.8	1.17	28.8
Dutch white clover	0.56	32.9	11.52	0.746	38.1	2.58	14.8	30.9	1.76	17.5
Alsike clover	0.71	27.4	9.83	0.694	40.9	2.94	13.9	31.1	2.15	14.5
Flame crimson clover	0.22	18.9	7.01	0.436	38.0	2.43	15.6	32.6	1.66	19.7
Common vetch	0.19	16.4	6.47	0.392	41.0	2.51	16.9	37.4	1.57	23.6
Winter peas	0.39	25.7	10.00	0.734	41.4	2.84	14.6	32.1	2.81	11.4
Spring lentil	0.20	9.5	3.54	0.180	39.3	1.99	19.7	33.4	1.69	19.6
Ladino white clover	0.56	34.5	11.91	0.807	38.5	2.68	14.4	30.0	1.88	15.7
Clover blend	0.55	29.4	11.13	0.804	38.8	2.96	13.1	36.3	2.31	15.8
Morton winter lentil	0.97	19.3	5.85	0.306	41.0	1.95	21.3	20.8	1.20	16.4
Winfred brassica	0.50	24.0	7.73	0.189	39.9	0.88	47.3	20.0	0.73	27.1
White mustard	0.39	11.8	4.22	0.156	39.9	1.46	27.4	26.6	0.99	26.0
Buckwheat	0.12	20.7	7.93	0.182	39.5	0.89	44.7	28.6	0.80	36.1
Common yarrow	0.88	13.0	4.64	0.181	35.8	1.92	19.1	37.2	0.95	39.1
**LSD**	**0.45**	**5.20**	**1.578**	**0.1011**	**4.04**	**0.374**	**5.52**	**12.44**	**0.523**	**5.03**
**Source of Variance**										
Block	ns ^1^	ns	ns	ns	ns	ns	ns	ns	ns	ns
Treatment	*** ^2^	***	***	***	**	***	***	ns	***	***

^1^ ns is not statistically significant. ^2^ ** and *** are statistically significant at *p* ≤ 0.001 and *p* ≤ 0.0001, respectively.

**Table 4:** Impact of various cover crop species on soil NH_4_^+^, NO_3_^−^, Mineral N, N Utilization Efficiency (NU_t_E), N Uptake Efficiency (NU_p_E), and NUE_soil_.

Plant Species	Soil N (mg N kg^−1^ Soil)			NU_t_E	NU_p_E	NUE_soil_	RANRI
	NH_4_^+^	NO_3_^−^	Mineral N	g g^−1^	%	g g^−1^	%
Fairway C. wheatgrass	1.27	4.03	5.30	91.8	11.2	103	79.1
Red fescue	2.05	5.49	7.54	73.8	13.0	93	62.1
Sheep fescue	2.82	19.2	22.02	--	--	--	-
Tall fescue	0.74	2.45	3.16	102.0	17.3	174	56.1
Indian ricegrass	2.73	20.6	23.32	--	--	--	-
Perennial ryegrass	1.47	4.01	5.49	90.0	16.2	144	54.3
Pubescent wheatgrass	0.95	3.84	4.79	86.3	17.3	149	52.5
Fall rye	1.59	3.97	5.57	123.3	21.7	267	40.4
Buffalo grass	1.42	6.49	7.91	70.9	9.3	63	85.5
Birdsfoot trefoil	1.63	3.73	5.36	79.1	24.4	190	36.2
Dutch white clover	1.69	4.18	5.87	44.1	78.8	347	11.0
Alsike clover	0.70	3.11	3.81	39.2	73.3	290	12.9
Flame crimson clover	1.97	3.04	7.47	43.4	46.1	200	17.4
Common vetch	2.45	6.69	9.42	43.6	41.5	173	17.6
Winter peas	1.48	3.72	5.64	35.7	77.6	271	11.3
Spring lentil	0.89	4.85	5.74	51.8	19.1	101	45.7
Ladino white clover	1.21	3.34	4.56	42.9	85.2	365	10.7
Clover blend	1.14	2.36	3.73	36.6	84.9	310	11.1
Morton winter lentil	0.78	4.24	5.01	69.0	32.3	203	27.7
Winfred brassica	0.92	3.89	4.81	127.4	20.0	253	45.3
White mustard	1.09	3.24	4.33	75.5	16.5	125	56.0
Buckwheat	0.84	3.49	4.33	113.6	19.3	219	48.0
Common yarrow	1.01	6.54	7.55	71.3	19.1	137	41.8
Control	2.56	26.03	28.59	--	--	--	--
**LSD**	**1.188**	**3.231**	**3.491**	**22.08**	**10.67**	**54.93**	**12.75**
**Source of Variance**							
Block	ns ^1^	ns	ns	ns	ns	ns	ns
Treatment	** ^2^	***	***	***	***	***	***

^1^ ns is not statistically significant. ^2^ ** and *** are statistically significant at *p* ≤ 0.001 and *p* ≤ 0.0001, respectively.

**Table 5:** Influence of various cover crop species and their residue incorporation on grapevine shoot C, N and C/N, lower (SPAD_L_) and upper (SPAD_U_) leaves SPAD, shoot dry matter (GSDM), and root fresh matter (GRFM).

Plant Species	Grapevine Shoot			SPADL	SPADU	Leaf Number	GSDM	RFM
	C (%)	N (%)	C/N					
Fairway C. wheatgrass	44.3	2.02	22.5	33.5	18.1	42	20.1	58.8
Red fescue	44.5	1.9	25.3	38.8	18.0	42	25.4	82.6
Sheep fescue	44.0	1.77	23.6	34.0	17.1	35	18.4	69.5
Tall fescue	44.5	1.63	28.2	28.5	16.6	36	27.4	70.8
Indian ricegrass	44.0	2.28	20.5	38.4	20.1	35	17.9	82.4
Perennial ryegrass	44.7	1.43	27.0	31.9	16.9	43	31.6	90.5
Pubescent wheatgrass	44.2	2.02	19.7	37.0	18.8	36	18.6	89.5
Fall rye	44.4	1.91	25.7	32.4	14.5	43	28.8	77.0
Buffalo grass	44.3	2.04	20.9	37.1	16.5	53	23.9	85.8
Birdsfoot trefoil	44.8	1.4	28.9	34.8	17.4	43	27.9	95.6
Dutch white clover	44.7	1.73	24.5	33.9	16.6	42	27.2	75.0
Alsike clover	44.2	1.84	25.0	33.9	15.6	44	24.7	88.6
Flame crimson clover	44.8	1.71	32.4	30.6	14.2	40	25.6	85.7
Common vetch	44.5	1.87	26.3	36.1	15.8	44	23.9	80.3
Winter peas	44.4	1.62	24.2	38.2	16.3	47	25.2	85.1
Spring lentil	44.5	1.93	22.1	32.9	16.1	44	29.5	65.3
Ladino white clover	44.4	1.74	25.0	37.3	19.3	57	27.9	80.0
Clover blend	44.8	1.27	31.8	32.5	13.7	43	32.1	72.4
Morton winter lentil	43.9	1.77	21.9	35.6	15.0	36	22.0	57.4
Winfred brassica	44.9	1.73	25.5	32.5	15.8	46	26.9	61.7
White mustard	44.7	1.79	24.9	33.5	16.3	41	31.6	60.3
Buckwheat	44.5	1.79	27.4	31.8	14.1	38	28.6	60.4
Common yarrow	44.8	1.82	24.6	36.7	14.6	35	30.0	51.0
Control	44.2	2.06	24.4	35.8	18.1	43	25.1	61.6
LSD	0.71	0.56	7.35	6.08	3.56	15.1	8.14	41.47
Source of Variance								
Block	ns ^1^	ns	ns	ns	** ^2^	*	*	ns
Treatment	ns	ns	ns	ns	ns	ns	*	ns

^1^ ns is not statistically significant. ^2^ *, ** are statistically significant at *p* ≤ 0.01 and *p* ≤ 0.001, respectively.

## Data Availability

Data are available upon request.
